# Kidney Transplantation From Hepatitis-C Viraemic Donors:Considerations for Practice in the United Kingdom

**DOI:** 10.3389/ti.2022.10277

**Published:** 2022-05-03

**Authors:** Daniel T. Doherty, Varinder Athwal, Zia Moinuddin, Titus Augustine, Martin Prince, David van Dellen, Hussein A. Khambalia

**Affiliations:** ^1^ Department of Renal and Pancreatic Transplantation, Manchester University NHS Foundation Trust, Manchester, United Kingdom; ^2^ Faculty of Biology, Medicine and Health, University of Manchester, Manchester, United Kingdom; ^3^ Department of Hepatology, Manchester University NHS Foundation Trust, Manchester, United Kingdom

**Keywords:** kidney transplant, hepatitis C infection, viraemia, donor, utilisation

## Abstract

**Background:** Donor hepatitis-C (HCV) infection has historically represented a barrier to kidney transplantation (KT). However, direct-acting antiviral (DAA) medications have revolutionised treatment of chronic HCV infection. Recent American studies have demonstrated that DAA regimes can be used safely peri-operatively in KT to mitigate HCV transmission risk.

**Methods:** To formulate this narrative review, a comprehensive literature search was performed to analyse results of existing clinical trials examining KT from HCV-positive donors to HCV-negative recipients with peri-operative DAA regimes.

**Results:** 13 studies were reviewed (11 single centre, four retrospective). Outcomes for 315 recipients were available across these studies. A sustained virological response at 12 weeks (SVR12) of 100% was achieved in 11 studies. One study employed an ultra-short DAA regime and achieved an SVR12 of 98%, while another achieved SVR12 of 96% due to treatment of a missed mixed genotype.

**Conclusion:** HCV+ KT is safe and may allow increased utilisation of organs for transplantation from HCV+ donors, who often have other favourable characteristics for successful donation. Findings from US clinical trials can be applied to the United Kingdom transplant framework to improve organ utilisation as suggested by the NHSBT vision strategy *“Organ Donation and Transplantation 2030: meeting the need”.*

## Background

Historically, donor infection with hepatitis-C virus (HCV) has been a barrier to kidney transplantation (KT). This was due to concerns regarding HCV transmission in the context of immunosuppression (IS) with reports of rapidly progressive liver disease in cases of inadvertent viral transmission or glomerulonephritis, directly damaging the implanted kidney (1). Furthermore, interferon therapies, the previous mainstay of HCV treatment were linked with organ rejection (2). Developments of novel antiviral therapeutic agents over the past decade, however, are beginning to change the landscape of transplantation.

The development of direct-acting antiviral medications (DAA) have revolutionised care of management of chronic HCV infection. Once-daily oral regimens varying between 8 and 16 weeks are very well tolerated and have shown efficacy of >95% of a sustained virological response at 12-weeks (SVR12), indicating viral clearance and cure (3). In times of increased organ demand, such developments have opened the door to a previously overlooked donor pool. Between 2005 and 2014, 3273 HCV antibody positive donors were identified in the United States. Only 37% of retrieved kidneys from this group proceeded to transplantation, the overwhelming majority in HCV-positive recipients. From this group, 4,144 kidneys were discarded although, other than HCV infection, they displayed favourable donor characteristics defined by Kidney Donor Profile Index (KDPI). Moreover, the public health crisis of non-prescribed opioid use in North America has seen a surge in deaths in intra-venous drug users under the age of 50 years old many of whom are HCV-positive and who otherwise might be considered for organ donation (4,5). As a consequence of this, the demographics of potential HCV-positive donors have altered, with the median age decreasing from 47 in 2012 to 35 in 2016 (4). Consequently, if HCV risks can be mitigated, there is the opportunity to increase the donor pool with organs with favourable characteristics for organ transplantation.

These epidemiological changes mean that consideration of HCV-positive donors will become a more commonplace scenario for the transplant clinician. Here, we will discuss how strategies have evolved to mitigate peri-transplant HCV transmission and consider how these developments which have been driven by necessity in North America can be applied to improve utilisation of organs for safe KT within the United Kingdom transplant setting.

## Methodology

A comprehensive database search was performed to formulate this narrative review of the literature. Search strategies employed MedLINE, EMBASE and Cochrane databases to identify studies published up to December 2021. Searches were performed for English language texts using MeSH terms “Kidney Transplantation” AND “Hepatitis C” AND “Tissue Donors”. These terms were also used as keywords within searches. All subsequent abstracts were reviewed. Articles relating to treatment of chronic recipient HCV infection, inadvertent HCV transmission, KT in HIV/HCV co-infection, simultaneous liver-kidney transplantation and HCV+ to HCV- KT prior to the DAA era were excluded. Published articles demonstrating the use of DAA interventions to mitigate the risk of HCV transmission were included. Both prospective and retrospective studies were included. References from the identified studies were also explored to highlight additional studies. United Kingdom transplant data was taken from publicly available annual reports produced by NHS Blood and Transplant and published literature.

## Direct-Acting Antiviral Therapy and HCV-Positive Kidney Transplantation

### HCV-Positive Testing and Definitions

Review of early studies of HCV positive donors may be confounded by changes in definition of HCV positivity. Historical and very early studies classed donors as HCV positive based on the presence of anti-HCV antibodies. The more widespread application of HCV antigen test with nucleic acid testing (NAT), by assessing viral RNA by polymerase chain reaction (PCR), allows the detection of contemporaneous viraemia. However approximately 25% of HCV antibody positive individuals will not be chronically infected and thus not viraemic due to spontaneous (innate) viral clearance (6), with a very low to no transmission risk. Furthermore, the roll out of therapeutic and public HCV elimination strategies means an increasing proportion of previous infected individuals will have now been cured of their infection. It is now consensus, that HCV-positive status, should be defined as the presence of HCV NAT viraemia, which conveys risk of transmission. Therefore, it is essential that chronic infection is defined based on detection of HCV NAT. It should also be noted, that immediately following HCV exposure, there is thought to be a window of up to 7 days in which viraemia may be present, but NAT will be negative. This is termed the eclipse window (4).

### HCV+ to HCV+ Kidney Transplantation

DAA regimes have been applied successfully to KT in HCV-positive recipients in a number of North American centres. Outcomes of 40 HCV-positive recipients were examined retrospectively, 19 of whom received an HCV-positive KT. Twenty-three received Ledipasvir (LDP) and Sofosbuvir (SOF), 12 received SOF and Simeprevir (SIM) and four received LDP, SOF and Ribavirin (RIB) in combination. Thirty-six patients received 12 weeks of DAA therapy, while the remainder received 16 or 24 weeks, as directed by a transplant hepatologist. All patients achieved a sustained virologic response at 12 weeks (SVR12) with good tolerance of treatment and 100% 1 year graft survival (7). This successful approach has been echoed in another cohort of 25 HCV-positive recipients who received an HCV-positive KT, with the majority receiving a 12-week DAA regimen, initiated at a median of 125 days (IQR 100–169) post-transplant. One recipient was non-compliant, producing an intention to treat derived SVR12 of 96% (8). Critically, both of these studies noted a reduced time on the waiting list after acceptance of an HCV-positive KT (7,8). For these recipients, the developments in DAA regimens, mitigated HCV risk and was favourable when compared to a prolonged period on dialysis with its associated morbidity and mortality. These initial studies have demonstrated how recipients can benefit from the safe expansion of the donor pool with good outcomes which has now become established practice. Such studies have also encouraged other investigators to consider the safe use of HCV-positive kidneys in HCV-negative recipients.

### HCV+ to HCV− Kidney Transplantation

Several centres have made significant progress in this field over the past 5 years (Table 1). Initial studies used 12 week regimens of Gazoprevir (GZR) and Elbasvir (EBR) in small single centre prospective cohorts to good effect, demonstrating 100% SVR12 (*n* = 10 and 10 respectively) (9,10). These studies used majority DBD (100% and 80%) donors with median ages [30 (IQR 23–35) and 31 (IQR 29–42)], demonstrating the advantageous demographics previously described in HCV-positive donors (5). Different timepoints for the onset of DAA regimens were used by these study groups. In the THINKER trial, Goldberg et al (9) initiated the DAA regime on post-transplant day 3 after HCV viraemia had been detected within the transplant recipients, whereas Durand et al (10) opted for a pre-emptive approach in EXPANDER. This initiated DAA therapy immediately post-transplant. These two strategies of transmit and treat versus prophylactic regimens have been mirrored in subsequent generations of peri-transplant DAA studies. As DAA studies in this field have emerged as successful and safe, investigators have sought to determine the optimal course timing and duration, without sacrificing efficacy (11).

**TABLE 1:** Studies investigating HCV+/HCV- kidney transplant with DAA regimes.

Author	Sample	Donor	Recipient	Genotypes	Immunosuppression	DAA regime	SVR	Notes
Durand et al 2017 (10)	*n* = 10	100% DBD	20% female	G1a 30%	Induction: Methylprednisolone, rATG	G1a: GZR/EBR 12/52	100% at 12/52	Pre-emptive
EXPANDER	Single centre	Median 30yo (IQR 23–35)	Median 71yo (IQR 65–72)	G2 10%	Maintenance: Tacrolimus, MMF, Prednisolone	G2 and G3: GZR/EBR + SOF 12/52		No DAA SAE
	Prospective	KDPI 45% (IQR 32–48)	Pre-transplant dialysis 1.6 years (IQR 0–2.6)	G3 10%				
	Non-randomised			G1a/3 10%				
				Indeterminate 40%				
Goldberg et al 2017 (9)	*n* = 10	80% DBD	50% female	G1a 100%	Induction: Methylprednisolone, rATG	GZR/EBR 12/52	100% at 12/52	DAA started after HCV viraemia detected POD3
THINKER	Single centre	Median 31yo (IQR 29–42)	Median 59yo (IQR 52–63)		Maintenance: Tacrolimus, MMF, Prednisolone			1 case possible DAA FSGS
	Prospective	KDPI 42% (IQR 32–48)						
	Non-randomised							
Molnar et al 2019 (32)	*n* = 53	89% DBD	18% female	G1a 64%	Induction: rATG	89% GLP/PTR	100% at 12/52	DAA started after HCV viraemia 4–8/52 post KTx
	Single centre	Mean 32.2yo (SD ± 5.3)	Mean 52.6yo (SD ± 10.9)	G1b 2%	Maintenance: Tacrolimus, MMF, Prednisolone	9% SOF/VPT		DAA AE due to delayed treatment
	Retrospective			G2 6%		2% SOF/LDP		
				G3 28%				
Friebus-Kardash et al 2019 (29)	*n* = 7	57% female	57% female	G1a 28%	Induction: Basiliximab	G1a: SOF/VEL or SOF/VEL/RIB	100% at 12/52	DAA started after recipient viraemia detected; median POD7
	Single centre	Mean 44.2 yo (SD ± 10.2)	Mean 52.8yo (SD ± 13.5)	G1b 42%	Maintenance: Tacrolimus, MMF, Prednisolone	G1b: SOF/LED		No DAA SAE
	Retrospective			G3a 28%	1 used anti-CD40 Ab for induction in place of Tac; 1 received plasmapheresis and IVIG due to HLA pre-sensitisation	G3a: SOF/VEL (All 8–12/52)		
Gupta et al 2019 (17)	*n* = 50	KDPI 62% (SD ± 18)	36% female	G1a 19%	Induction: rATG	Prophylaxis 2–4/7 SOF/VEL	98% at 12/52	Pre-emptive
	Single centre		Median 60yo (IQR 36–76)	G2 4%	Maintenance: Tacrolimus, MMF, Prednisolone	If HCV transmission ELB/GZR 12/52 + 2nd line option if required		Ultra-short course promotes DAA resistant HCV mutations
	Adaptive trial design			G3 12%				
				Indeterminate 8%				
				Unknown 8%				
Duerr et al 2019 (15)	*n* = 7	Mean 46.4 (±SD 7.8)	Mean 59.4 (±SD 8.4)	G2a 100% (of those NAT+)	Induction: Basiliximab	DCV/SOF 12/52	100% at 12/52	Pre-emptive
	3 HCV NAT+, 4 HCV Ab +				Maintenance: Tacrolimus, MMF, Prednisolone			Seroconversion (HCV Ab+) at 12/52 in 5/7 recipients
	Single centre							
	Prospective							
Kapila et al 2020 (34)	*n* = 64	Median 32 (range 19–56)	Median age 69.5 (range 32–81)	G1 5%	Induction: Methylprednisolone, rATG	LDP/SOF 12/52 37.5%	At end of study period	DAA started after viraemia (median 72 days; range 9–198)
	Single centre	KDPI 54% (range 25–99)	68.8% male	G1a 59%	Maintenance: Tacrolimus, MMF, Prednisolone	GLP/PTR 12/52 51.2%	58 received DAA	3 patients did not develop viraemia
	Prospective			G2 9%		VEL/SOF 12/52 1.6%	41 SVR12	2 cases FCH
				G3 13%			10 HCV NAT- but had not reached 12/52 follow up 7 DAA current treatment	
				G4 5%			1 case of resistance with prolonged therapy due to resistance	
				Mixed 5%				
Sise et al 2020 (12)	*n* = 30	KDPI 53% (IQR 41–65)	30.0% female	G1a 43%	Usual standard of care	GLP/PTR 8/52	100% at 12/52	DAA started POD 2–5
MYTHIC	Multicentre	Median 33.5yo (IQR 29–38)	57yo (IQR 51–60)	G2 3%	Variation of regimes between centres			Median 6-month eGFR 57 ml/min/1.73 m^2^
				G4 3%				No DAA SAE
				Unknown 50%				
Sise et al 2020 (13)	*n* = 8	100% DBD	25% female	G1a 100%	Induction: Methylprednisolone, rATG	GZR/ELB 12/52	100% at 12/52	Pre-emptive
	Single centre	Median 27yo (IQR 25–30)	Mean 55.9yo (SD ± 9.4)		Maintenance: Tacrolimus, MMF, Prednisolone			No DAA SAEs
	Retrospective	KDPI 31% (IQR 29–65)						
Feld et al 2020 (20)	*n* = 30	Median 36 (IQR 31–39)	77% male	G1 50%	Usual standard of care	EZE (10 mg) + GLP/PTR (300mg/120 mg) 7/7	100% at 12/52	Pre-emptive
	Single centre		Median 61yo (IQR 48–66)	G2 11%	Cyclosporin avoided			1 DAA serious AE (transient elevation of liver enzymes in KT recipient)
	Heterogeneous recipients (10 KT, 1 SPK)			G3 28%				
				Unknown11%				
Jandovitz et al 2020 (16)	*n* = 25	Mean age 35yo (SD ± 8.9)	76% male	G1a 60%	Induction: Basiliximab	LDP/SOF 12/52 56%	96% at 12/52	DAA start median 13 days (IQR 8–22)
	Single centre	KDPI 49 (IQR 38–66)	Mean age 57.7yo (SD ± 10.4)	3a 28%	Maintenance: Tacrolimus, MMF, Prednisolone	VEL/SOF 12/52 32%	1 case of mixed genotype requiring re-treatment to achieve SVR12	
	Retrospective							
Durand et al 2020 (14)	*n* = 10	Median age 38.5yo (IQR 20–45)	70% male	G1a 60%	Not specified	GLP/PTR 4/52	100% at 12/52	Pre-emptive
	Single centre	KDPI 60% (29–76)	Median 67yo (IQR 40–75)	G1b 10%				No DAA SAE
				G3 20%				
				Unknown 10%				
Terrault et al 2021 (46)	*n* = 24	Median age 36 (IQR 31–41.5)	KT recipients 45% male	Not specified	Usual standard of care	SOF/VEL 12/52	100% at 12/52	DAA start median 16.5 days (IQR 9.8–24.5)
	Multi-centre	KDPI (52 (40.5–61.5)	Median age 54 (IQR 52–57)					No DAA SAE in KT group
	Heterogeneous recipients (11 KT)							

Ab—antibody; AE—adverse event; DAA—direct acting antiviral; DCV—daclatasvir; ELB—elbasvir; EZE—ezetimibe; FCH—fibrosing cholestatic hepatitis; GLP—glecaprevir; GZR—grazoprevir; HCV—hepatitis C virus; HLA—human leukocyte antigen; IQR—inter-quartile range; IVIG—intravenous immunoglobulin; KDPI—kidney donor profile index; KT—kidney transplant; LDP—ledipasvir; MMF—mycophenolate mofetil; NAT—nucleic acid amplification test; POD—post-operative day; PTR—pibrentasvir; rATG—rabbit antithymocyte globulin; SAE—serious adverse event; SPK—simultaneous kidney pancreas transplant; SIM—simperavir; SOF—sofosbuvir; Tac—tacrolimus; VEL—velpatasvir.

Early studies favoured testing for HCV genotype with subsequent genotype specific treatment, whereas, more recently, small volume studies have used pangenotypic agents for long or intermediate post-transplant durations and achieved satisfactory results (Table 1) (12–14). These have mostly been used in confirmed cases of HCV NAT+ donors, but one strategy has employed the use of pangenotypic DAAs in HCV NAT- Ab+ donors in addition (15). Of note, transmit and treat strategies which do not employ pangenotypic agents are reliant on accurate genotyping, this can cause difficulty when mixed genotypes are not detected (16). Gupta et al (17) used an adaptive trial design to trial two to four doses of pangenotypic SOF and Velpatasvir (VEL) on transplant day 0–4. This was commenced immediately pre-transplant to prevent transmission in 50 recipients. Six cases across all phases of the study required 3 months of DAA treatment for HCV transmission. This regimen was associated with a lower SVR12 compared to other trials (98%), and three recipients of six cases of HCV transmission developed treatment resistant mutations. One recipient also developed acute rejection simultaneously to developing HCV viraemia, which the authors suggest could have contributed a non-specific immune response triggering rejection. Given the inferior results in comparison to widespread success with longer DAA regimes, the authors suggested that this course length should not be adopted. While such an approach may be favoured by healthcare funders, the outcomes appear inferior.

Following the use of a 4-week course of SOF/VEL producing 100% SVR12 in a cohort of 44 cardiothoracic transplant recipients (36 lung, 8 heart) receiving organ from HCV+ donors without any adverse events, a similar strategy has been applied to kidney transplantation (18). Durand et al (14) used GLP/PTR combination therapy for 4 weeks with the first dose administered prior to organ perfusion. This small study demonstrated feasibility of such an approach in renal transplant recipients with a 100% SVR12. HCV was transmitted in 50% of cases, of which all had undetectable levels of HCV RNA 2 weeks after treatment was commenced. This strategy, although only a preliminary study, seems to balance the safety requirements required with excellent efficacy and a short duration, making prophylactic regimens acceptable for healthcare funders. It should be noted that DAAs have been well tolerated in all transplant studies to date as has been described in the literature relating to treatment for chronic HCV. In particular, toxicity is infrequent and not severe, not usually requiring treatment alteration and there are few drug-drug interactions (DDI), which is of special importance in the transplant cohort (19). Of note, cyclosporin has been avoided in previous trials due to DDI risk due associated with GLP and GZR, but is no longer generally favoured for use in immunosuppressive regimes (20,21). As such, review by pharmacist with experience in management of HCV DAAs is of importance.

The shortest regime has been applied to a heterogeneous group of 30 transplant recipients with success (10 KT, one simultaneous pancreas and kidney (SPK)) (20). In addition to a DAA regime of GLP/PTR, Ezetimibe (EZE) was also administered with eight doses (one prior to transplant and on seven subsequent post-transplant days). EZE acts as a Niemann-Pick C1-like 1 (NPC1L1) receptor antagonist, a key component of cholesterol uptake in hepatocytes, warranting its use in hypercholesterolaemia. NPC1L1, is also targeted by HCV for hepatocyte cell entry and has been demonstrated to block this *in vitro* and reduce HCV establishment of some genotypes *in vivo* mouse models (22). 67% of recipients developed transient viraemia, with HCV RNA undetectable by 14 days post-transplant and 100% SVR12. This initial transmission rate is comparable with other studies, without the use of EZE, suggesting that its role needs further investigation. These studies have changed the field, but more is required to facilitate widespread use outside of clinical trials. The studies to date are published by single specialist centres with small sample sizes and only limited follow up with regards to graft function. Many of the studies have heterogeneous organ recipients. This should be considered, as although useful for demonstrating initial safety and proof of principle, there may be important factors to observe in longer term follow up between organ recipient groups and different immunotherapeutic regimens. Treatment resistance emerged as a concern following short course DAA regimes. This is something which should be monitored closely in other larger and longer-term studies to examine whether this phenomenon also is exhibited in longer DAA regimens but has not been detected due to insufficient study power. Given the risks of chronic HCV infection to the recipient in the advent of failed viral clearance, a low threshold for treatment failure should be established in future studies and clinical practice. Reassuringly, no variation in standard immunosuppressive regimes have been employed in the existing trials to date (Table 1), such requirements would represent significant concerns for transplant clinicians and any requirement for immunosuppression alterations should be recorded in future trials and registries. Currently, kidney transplantation in the context of HCV has been performed in small volumes at a limited number of centres and more comprehensive data is not currently available.

## United Kingdom Landscape

The utilisation of organs from HCV positive donors is not established practice in the United Kingdom and the prevalence of HCV positive donors is lower than in North American populations. Between 2010 and 2014 of 8,184 potential organ donors with acquired consent for transplantation, 77 tested anti-HCV antibody positive: a prevalence of 0.94% (CI 0.74–1.18). 54 of this group were below the age of 54 with 42 having injected recreational drugs of which 21 had continued active use (23). This represents a lower volume than the United States but mirrors the typically younger age of HCV+ donors.

In 2018, 26 individuals where identified, and consent acquired for donation who also tested positive for anti-HCV antibody. Of these 26 patients, only five had organs utilised. In 2019, 50 anti-HCV antibody positive donors were identified and consented, but only 16 of these proceeded to donation. Exact reasons for this attrition are not specified but it is presumed to be due to concerns regarding transmission. Unfortunately, data on organ specific patterns are not available. The median ages of those that proceeded to donation in those years were 41.9 and 44 years, respectively, lower than the mean age of all donors of 52, demonstrating the possible benefits in utilisation (24,25). Of note, although younger than the mean United Kingdom age, this is older than the typical age seen in HCV+ donors in the United States (4).

Mitigating the risk of HCV transmission would allow a greater proportion of these donors to proceed to donation and increased organ utilisation. In 2018–2019, this would equate to a potential of 76 donors and 152 kidney recipients. Of note, HCV RNA screening is not routine for potential United Kingdom donors. Consequently, an unknown proportion of these donors may not have been HCV viraemic at time of donation, a scenario which has been demonstrated to be safe in some cohorts (26,27). The addition of HCV NAT testing in the United Kingdom, would allow improved objective assessment to allow transmission risk to be considered and potentially mitigated. Although this article analyses epidemiological factors within the United Kingdom, we anticipate that this is similarly applicable to other European populations where opiate use is less prevalent than the United States; indeed promising early German and Spanish experiences have been published (28,29).

## Future Considerations for United Kingdom Application

As discussed, there has been continued advance in DAA therapy to mitigate transmission from HCV viraemic donors, which has allowed increased organ utilisation in the US. The most recently published data by NHSBT in the United Kingdom suggests that there is a potentially under-utilised donor pool within the United Kingdom. Consequently, the increased use of such organs should be considered, resulting in significant benefits for patients on the transplant waiting lists. The joint United Kingdom vision statement “Organ Donation and Transplantation 2030: Meeting the need” highlights the need to further increase organ utilisation. Instigation of recommendations from “Taking Organ Transplant to 2020” has led to an increase in the successful utilisation of older donors with more comorbidities with sustained level of outcomes nationally but opportunities remain for improvement (30). Although the number of people waiting for a kidney transplant in the United Kingdom had reduced to 2015, since then the number on the active waiting list for a cadaveric kidney transplant has plateaued around 5,000 patients (2017/18: 5,033; 2018/19: 4,977: 2019/20: 4,960), 67% of whom are still waiting beyond a year for transplantation (31). As we have described, although HCV+ positive donors have been identified, the proportion of organs utilised could be improved. Consequently, as the waiting list continues to build, utilisation of HCV+ organs with DAA regimens to mitigate transmission risk represents a feasible and sustainable strategy to achieve the goals for 2030.

Real world data from the US has demonstrated that outside of clinical trials, where regimens are supplied by manufacturers or funding for DAA therapy is guaranteed, there have been difficulties in acquiring approval from insurers following HCV transmission (16,32). Many funders are reluctant to provide cover for a pre-emptive or prophylactic DAA regimen and subsequently favour transmit and treat approaches (33). Consequently, this has led to delays in treatment (34). Such delays have the potential to induced sequelae of HCV infection, with serious implications such as fibrosing cholestatic hepatitis (35). It should be noted that treatment failure has the potential to induce devastating complications including graft loss. Concerns have also been raised regarding the increased risk of the development of BK viraemia and cytomegalovirus (CMV) and severe cases have coincided with the formation of *de novo* donor specific antibodies (32,36). Studies to date have not noted significant difference in the prevalence of such viral complications, but when such events occur, the severity has been increased (36,37). Consequently, thorough surveillance strategies will be required.

National funding strategies on medication approval based on evidence-based medicine and controlled by the National Institute for Clinical Excellence removes this consideration from the equation in the United Kingdom. As a result, prophylactic regimens which can be approved for patients nationally may be more palatable in the United Kingdom and may mean that translation from clinical trials to common practice is less challenging. The possibility of short course DAA regimens make this even more possible. From a health economics perspective, the potential to reduce waiting list time and associated long term dialysis costs are likely to offset the cost of DAA regimens, making such strategies appealing when overall cost of care for patients with ESRD are considered. The unit price for a 28 day pack of GPR-PBR is £12,993.99 as reported by NICE for use in chronic HCV (38), while estimated annual dialysis costs in the United Kingdom are £24,043 and £20,078 for haemodialysis and peritoneal dialysis respectively (39). This has been robustly demonstrated in the Canadian and US populations and agreed by the United Kingdom joint taskforce (40,41). The cost benefits for providers will also be greater if short courses of DAA regimens as described by Durand et al (14) and Feld et al (20) can become standard care.

Despite the evidence of safety, patient perception and education regarding this novel approach is paramount. HCV for many has an associated stigma and may result in reduced uptake. However, several studies have shown that those in receipt of an HCV+ transplant have had positive experiences. For most recipients surveyed, the benefit of reduced waiting list time was important in their choice to accept an HCV+ organ. Smaller numbers reported concerns with donor lifestyle factors and a possibility that the organ they received was of lower quality and in one survey, only 9% were concerned about sexual transmission to partners although reported behavioural change, such as avoiding sharing glasses, due to concerns of transmission (42,43). In the follow up to the EXPANDER study, no patients report being victims of stigma or being treated differently and did not regret their involvement (44).

Despite the increasing amount of evidence, this remains a novel approach to care and warrants stringent observation and assessment in line with IDEAL standards (45). Through NHSBT the United Kingdom has excellent tools in place for clinical governance and registration with continued assessment of patient outcomes which is crucial as this option remains a treatment strategy which should be conducted within an investigative framework. Patients who choose to enrol in such schemes should be provided with sufficient information regarding the evidence to date including the possible consequences in order that informed consent can be acquired. In some non-publicly funded healthcare settings, it should also be necessary to determine the availability of DAA therapy prior to proceeding to transplant.

## Conclusion

There has been rapid progress in the development of DAA therapy after renal transplantation to facilitate the use of HCV viraemic donor organs safely in HCV non-viraemic recipients. Such strategies have been demonstrated to be safe in US clinical trials, but there have been difficulties in transforming this to become standard care. Although less than in North America, there is a potential pool of young, otherwise healthy donors with preferential characteristics for organ utilisation, if HCV transmission can be mitigated. The national funding and governance structure of United Kingdom healthcare allows evidenced based practice to be initiated with stringent assessment of outcomes to use this potential donor pool to safely reduce waiting list time for the benefit of all patients with ESRD.
